# Synthesis of polysiloxane elastomers modified with sulfonyl side groups and their electromechanical response†

**DOI:** 10.1039/d3tc00200d

**Published:** 2023-05-18

**Authors:** Yauhen Sheima, Thulasinath Raman Venkatesan, Holger Frauenrath, Dorina M. Opris

**Affiliations:** a Laboratory for Functional Polymers Swiss Federal Laboratories for Materials Science and Technology Empa Überlandstrasse 129 Dübendorf CH-8600 Switzerland Dorina.opris@empa.ch; b Institute of Chemical Sciences and Engineering Ecole Polytechnique Federale de Lausanne (EPFL) Station 6 Lausanne CH-1015 Switzerland

## Abstract

Dielectric elastomer transducers are elastic capacitors that respond to mechanical or electrical stress. They can be used in applications such as millimeter-sized soft robots and harvesters of the energy contained in ocean waves. The dielectric component of these capacitors is a thin elastic film, preferably made of a material having a high dielectric permittivity. When properly designed, these materials convert electrical energy into mechanical energy and *vice versa*, as well as thermal energy into electrical energy and vice versa. Whether a polymer can be used for one or the other application is determined by its glass transition temperature (*T*_g_), which should be significantly below room temperature for the former and around room temperature for the latter function. Herein, we report a polysiloxane elastomer modified with polar sulfonyl side groups to contribute to this field with a powerful new material. This material has a dielectric permittivity as high as 18.4 at 10 kHz and 20 °C, a relatively low conductivity of 5 × 10^−10^ S cm^−1^, and a large actuation strain of 12% at an electric field of 11.4 V μm^−1^ (0.25 Hz and 400 V). At 0.5 Hz and 400 V, the actuator showed a stable actuation of 9% over 1000 cycles. The material exhibited a *T*_g_ of −13.6 °C, which although is well below room temperature affected the material's response in actuators, which shows significant differences in the response at different frequencies and temperatures and in films with different thicknesses.

## Abstract 2

Polysiloxane elastomers modified with sulfonyl groups show a glass transition temperature of −13.6 °C, influencing the electromechanical actuation at different frequencies and temperatures.

## Graphical abstract

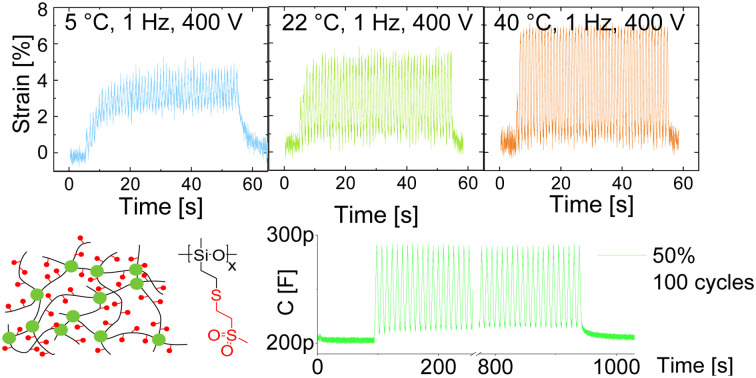

## Introduction

Technologies as different as artificial muscles, transducers, actuators, and capacitive sensors have in common that they all can be constructed from thin dielectric elastomer (DE) films.^1,2^ Two ultrathin electrodes applied on DE surfaces form dielectric elastomer transducers (DETs). They elongate and contract like natural muscles when electrically or mechanically stressed.^3^ DETs gained increasing importance in soft robotics,^4^ a technology with an estimated market value of 1 billion USD in 2020.^5^ Additionally, the industry extensively explores their use in wave energy harvesting. However, the potential of these devices goes beyond actuators, sensors, or generators.^6,7^ They could also be used, for example, in solid-state refrigeration and thermal energy harvesting.^8,9^ High dielectric permittivity improves the performance of actuators, sensors, and generators.^9^ It significantly reduces the driving voltage of dielectric elastomer actuators (DEAs), increases sensor sensitivity, and generally allows for more energy to be harvested at lower voltages. However, common elastomers such as silicones, polyurethanes, polyacrylates, or polybutadiene have a low dielectric permittivity.^10–14^ Two strategies have been followed to enhance this key property by introducing highly polarizable fillers or chemically modifying the elastomer with polar groups.^15–26^ The latter approach allows for synthesizing elastomers homogenous at the molecular level that have a long lifetime, high dielectric breakdown, and no Mullins effect.^27–31^

Recently, a systematic investigation on how different types and contents of polar pendant groups to the polysiloxane chain influence the dielectric properties and *T*_g_ has been reported by our group.^16,32,33^ This investigation eventually allowed us to find a polysiloxane modified with 2-(methylsulfonyl)-ethanethiol, which has a permittivity of about 22 at room temperature and high frequencies, as well as a *T*_g_ that is approaching 0 °C. While this rather high *T*_g_ may be detrimental for some applications, it also allows investigation of how such materials behave under an electric field in the vicinity of *T*_g_, where it may influence the electromechanical response. Such investigations have never been reported before, as only a few dielectric elastomers with a *T*_g_ slightly below room temperature are available.^34^ Therefore, polysiloxane elastomers modified with sulfonyl groups may show intriguing properties.

This work reports on the synthesis of polysiloxane elastomers modified with sulfonyl groups and their mechanical, dielectric, and electromechanical response at different electric fields, frequencies, and temperatures.

## Experimental

### Materials

Unless otherwise stated, all chemicals were of reagent grade and used without purification. 1,3,5,7-Tetramethyl-1,3,5,7-tetravinyl cyclotetrasiloxane (V_4_) was purchased from ABCR. 2,2-Dimethoxy-2-phenylacetophenone (DMPA), 2,2′-(ethylenedioxy)diethanethiol (CL2), pentaerythritol tetrakis (3-mercapto propionate) (CL4), benzene, toluene, sodium hydroxide, tetramethylammonium hydroxide 25% in MeOH (TMAH), hydrochloric acid, and thioacetic acid were purchased from Merck. Methanol, dimethylsulfoxide (DMSO), acetonitrile (ACN), and tetrahydrofuran (THF) were purchased from VWR. As a sacrificial layer, a solution of PVA in isopropanol/2-butanol from Suter Kunststoffe AG was used. Elastosil films with a thickness of 200 μm were purchased from Wacker. Polymethylvinylsiloxane (PV) (*M*_n_ = 105 500 g mol^−1^, *M*_w_ = 375 000 g mol^−1^, PDI = 3.55) and 2-(methylsulfonyl)-ethanethiol were prepared according to the literature.^32^

### Characterization

^1^H and ^13^C NMR spectra were recorded at 298 K on a Bruker Avance 400 NMR spectrometer using a 5 mm broadband inverse probe at 400.13 and 100.61 MHz, respectively. Chemical shifts (*δ*) in ppm are calibrated to residual solvent peaks (CDCl_3_: *δ* = 7.26 and 77.16 ppm). Size-exclusion chromatograms were used with an Agilent 1100 Series HPLC (Columns: serial coupled PSS SDV 5 μm, 100 Å, and PSS SDV 5 μm, 1000 Å; detector: DAD, 235 nm and 360 nm; refractive index), with THF as the mobile phase. PDMS standards were used for the calibration and toluene as an internal standard. As a UV source, a Hönle UVA HAND 250 GS UV lamp was used. DSC investigations were undertaken on a PerkinElmer Pyris Diamond DSC instrument. Two heating steps and one cooling step with a heating and cooling rate of 20 °C min^−1^ in the temperature range of −90 to 50 °C were conducted per measurement under a nitrogen flow (50 ml min^−1^). The second heating step was considered for the evaluation of the *T*_g_. About 10 mg of the sample was weighted in aluminum crucibles shut with pierced lids. The tensile tests and the cyclic uniaxial tensile stress tests were performed on a Zwick Z010 tensile test machine with a crosshead speed of 50 mm min^−1^. Tensile test specimens with a gauge width of 2 mm and a gauge length of 18 mm were prepared by die-cutting. The strain was determined over the traverse moving sensor. The curves were averaged from three different samples per material using Origin software. The tensile modulus was determined from the slope of the stress–strain curves using a linear fit to the data points within 10% strain. Dynamic mechanical analysis was carried out on a RSA 3 DMA from TA Instruments. Stripes of 10 mm × 25 mm were measured under a dynamic load of 2 g, at 2% strain in the frequency range of 0.05–10 Hz at 25 °C and 65% humidity. Permittivity measurements were performed in the frequency range of 0.1 Hz to 1 MHz using a Novocontrol Alpha-A Frequency Analyzer. The VRMS (root mean square voltage) of the probing AC electric signal applied to the samples was 1 V. The samples were squeezed between two electrodes (diameter of 20 mm). The actuation strain was measured optically as the extension of the diameter of the electrode area *via* a digital camera, using an edge detection tool of a LabView program to detect the boundary between the black electrode area and the transparent silicone film. Circular electrodes (8 mm diameter) of carbon black powder were applied to each side of the film. A FUG HCL-35-12500 high-voltage source served as a power supply for actuator tests. For the thick actuators (above 80 μm), 5% of prestrain was applied. Thinner actuators prepared using the sacrificial PVA layer were not prestrained. Temperature-dependent actuation measurements were conducted using a tall Berzelius beaker in which the actuator fit, placed in a sand bath covering most of the outer beaker's wall, and heated on a heating plate. A thermometer verified the temperature. Actuation measurements were performed five minutes after reaching the desired constant temperature. The thickness of the film was measured using a micrometer gauge with an uncertainty of ±5 μm.

Sensors were manufactured using either PDMS or E-CL2-2 films, which were covered on both sides with cross-linked electrodes Elastosil® LR 3162A/B.^35^ The electrode preparation, as well as the construction of the sensors, was described in detail elsewhere. The sensors were tested using a traction sliding machine Zaber A-LSQ300A-E01 for stretching, while a Keithley DMM6500 multimeter was used for capacitance measurement.

### General synthesis of polysiloxane containing 95% 2-(methylsulfonyl)-ethanethiol and 3.3% of vinyl groups (PSu)

PV (10 g, 0.116 mol repeat units, 1 eq.), 2-(methylsulfonyl)-ethanethiol (15.43 g, 0.11 mol, 0.95 eq.), and DMPA (0.30 g, 1.16 mmol, 0.01 eq.) were dissolved in the mixture of ACN : THF (1 : 1.5, 250 ml). The reaction mixture was degassed three times using the freeze–pump–thaw technique and irradiated for 20 min with UV light by placing the UV lamp at a distance of 20 cm from the flask. The solution was concentrated using a rotary evaporator, and functionalized polymer PSu was purified three times by dissolving in ACN and precipitating it in methanol. Some amount of the solvent was removed using a rotary evaporator. Then, the concentration of the polymer was adjusted to 60 wt% using ACN.

### Formation of polar elastomers (E-CLx-Y)

Polymer PSu (1.67 g) was mixed with a certain amount of cross-linker CLx (for amounts, see Table 1) and DMPA (4.5 mg). The obtained mixture was centrifuged for 2 min at 6000 rpm to remove the air bubbles. Thick films (>80 μm) were cast directly on a Teflon substrate, while thin films were cast on a glass coated with a sacrificial PVA layer. Thick films were exposed to air for 30 minutes and then cross-linked by irradiation with UV light for 5 minutes. The formed films were dried for 12 hours in a vacuum oven at 60 °C.

**Table 1:** Amount of reagents used for the synthesis of E-CLx-Y and their Young's modulus (*Y*_10%_), average strain at break (*s*_av_), storage modulus (*E'*), and tan *δ*

Name	CL2^a^ [μl]	CL4^a^ [μl]	*Y*_10%_ [kPa]	*s*_av_ [%]	*E*′@0.05 Hz [kPa]	tan *δ*@0.05 Hz
E-CL2-1	25^b^		270	136	235	0.166
E-CL2-2	50^b^		551	91	382	0.082
E-CL2-3	100^b^		628	53	518	0.04
E-CL2-4	200^b^		318	114	238	0.1
E-CL4-1		58^b^	863	56	697	0.058
E-CL4-2		117^b^	1100	39	1005	0.026

a A 20 vol% solution of CL1 and CL2 in THF was used.

b Volume of CL1 and CL2 solutions in THF (μl) was added to PSu (1 g).

## Results and discussion

**Scheme 1:**
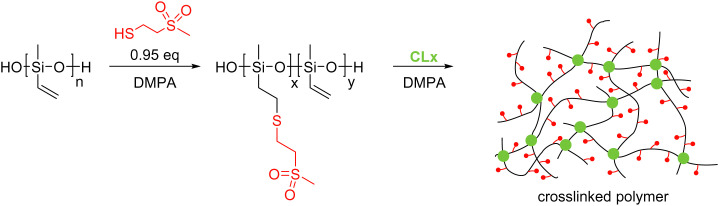
Synthesis of polysiloxanes PSu containing polar side groups and their cross-linking into elastomeric films.

Encouraged by the high room-temperature dielectric permittivity of about 22 at 10 kHz measured for a non-cross-linked polysiloxane modified at every repeat unit with a 2-(methylsulfonyl)-ethanethioether,^32^ here we explored the possibility of cross-linking it to elastomers with useful mechanical, dielectric, and electromechanical properties and their potential in different applications. The synthesis started from a polymethylvinylsiloxane (PV) (*M*_n_ = 105 500 g mol^−1^, *M*_w_ = 375 000 g mol^−1^, PDI = 3.55), which was reacted with 2-(methylsulfonyl)-ethanethiol (1) *via* the thiol–ene reaction (Scheme 1). Our previous attempts to synthesize a polysiloxane fully functionalized with polar thiol 1 had shown that the functionalized polymer was poorly soluble in THF.^32^ Therefore, the thiol–ene addition of thiol 1 to PV was conducted using a mixture of THF and DMSO. This mixture allowed for the dissolution of the starting PV and modified polymer PSu. Incomplete functionalization of PV using less than stoichiometric polar thiol to vinyl was successful, giving polymer PSu with around 3.3% unreacted vinyl groups (Scheme 1). Our attempts to remove the DMSO by vacuum distillation led to insoluble particles and the polymer solution turned dark. This may indicate that the vinyl groups of PSu were involved in an unwanted cross-linking reaction.

**Fig. 1:**
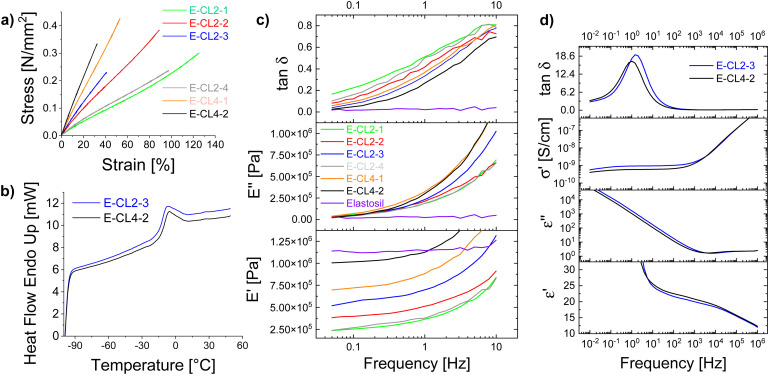
(a) Average stress–strain curves of elastomers E-CLx-Y. (b) DSC of samples E-CL2-3 and E-CL4-2. (c) DMA of materials E-CLx-Y in comparison with the Elastosil film. (d) Dielectric properties (permittivity (*ε*′), dielectric loss (*ε*′′), conductivity (*σ*′), and tan *δ*) as a function of frequency for the two most promising materials E-CL2-3 and E-CL4-2.

Therefore, the solvent used for the synthesis was replaced with acetonitrile. This allowed us to prepare PSu as a transparent yellowish solution after purification. The ^1^H NMR spectrum of PSu showed typical signals of the polar thioether groups and small signals at about 6 ppm for the vinyl groups (Fig. S1, ESI†). The obtained polymer was highly viscous and had to be cross-linked to achieve elastic materials. This was achieved using a thiol–ene reaction due to its versatility, reliability, and rate. Two different multifunctional thiols that function as cross-linkers CLx were explored: 2,2′-(ethylenedioxy)diethanethiol (CL2) and pentaerythritol tetrakis (3-mercapto propionate) (CL4). After mixing the PSu with a predefined amount of CLx and DMPA photoinitiator, the mixture was processed into thin films by doctor blading and cross-linked by irradiating with a UV light. The formed elastic materials were denoted as E-CLx-Y, where CLx stays for the cross-linker used and Y for different samples prepared. For the amounts of reagents used, see Table 1. The obtained materials were subjected to tensile tests and DMA. Fig. 1a shows the average stress–strain curves of at least three specimens obtained for the six synthesized materials E-CLx-Y (Fig. S2, ESI†) using Origin software; however, the values for strain at break in the graph represent the lowest value obtained for the different samples measured and not the average; therefore, these values were slightly smaller than those shown in Table 1, where the average is given. Materials E-CL2-1 to E-CL2-3 prepared using an increasing amount of CL2 showed a decrease in the strain at break from 136% to 53% and an increased Young's modulus from 270 kPa to 628 kPa (Table 1).

DMA measurements (Fig. 1c) showed that the storage modulus *E'* increased from 235 kPa for E-CL2-1 to 518 kPa for E-CL2-3, while the mechanical loss factor tan *δ* decreased from 0.166 for material E-CL2-1 to 0.04 for E-CL2-3 at low frequency. However, when a larger amount of CL2 was used, as was the case for E-CL2-4, a decrease in Young's and storage moduli and an increase in the tensile strain to 114% was observed, but tan *δ* at low frequencies increased to 0.1. By increasing the amount of CL2, the proportion of the thiol to vinyl groups increases and likely not all thiol groups are involved in the cross-linking and thus, CL2 is incorporated as dangling chains that function as plasticizers making the material softer. Additionally, the excess thiol can form disulfide bridges and increase the segment between cross-links and thus reduce the cross-linking density, making the material softer.

When CL4 was used instead of CL2 and the molar content of functional thiol to vinyl groups was kept constant, a drastic increase in Young's modulus for the latter was observed. Thus, materials E-CL4-1 and E-CL4-2 cross-linked using the same mole contents of thiol groups as E-CL2-2 and E-CL2-3, respectively, showed that Young's moduli increased from 551 to 863 kPa and from 628 to 1100 kPa. This indicates a higher cross-linking density in materials E-CL4-1 and E-CL4-2. A similar increase was observed for the storage modulus, while tan *δ* decreased to 0.058 for E-CL4-1 and 0.026 for E-CL4-2. For the latter, the losses at low frequency were similar to those of Elastosil, a commercial polydimethylsiloxane elastomer (tan *δ* = 0.018). However, materials E-CL4-Y showed a low strain at the break of 56% and 39% with increasing thiol content. Also, it should be mentioned that contrary to Elastosil, whose mechanical parameters were stable over a wide frequency range, the loss factor of materials E-CLx-Y showed a substantial rise with increasing frequency. This indicates the frequency-dependent nature of the materials with more pronounced viscoelastic behavior at higher frequencies, with values reaching 0.8, which is rather high. Two materials with the lowest mechanical loss factor at low frequency and better elastic properties were chosen for our subsequent investigations: E-CL2-3 and E-CL4-2. During our earlier investigations, we observed that materials with a mechanical loss factor below 0.05 were suitable for actuator applications, giving a fast and reversible response in electromechanical actuators.

The *T*_g_ of samples E-CL2-3 and E-CL4-2 was evaluated using DSC (Fig. 1b). As expected, due to the high polarity of sulfonyl groups, the *T*_g_ of the modified polymer approached 0 °C. Materials E-CL2-3 and E-CL4-2 had a *T*_g_ of −13.6 °C and −12.2 °C, respectively. These values were slightly higher than the reported *T*_g_ of the uncross-linked PSu,^32,33^ which is expected since the mobility of polymer chains decreases upon cross-linking. The thermal stability of the chosen samples was investigated by TGA. Fig. S3 and S4 (ESI†) show that both materials were stable up to 300 °C, while at 410 °C, about 48.5% of elastomer's weight was lost.

**Fig. 2:**
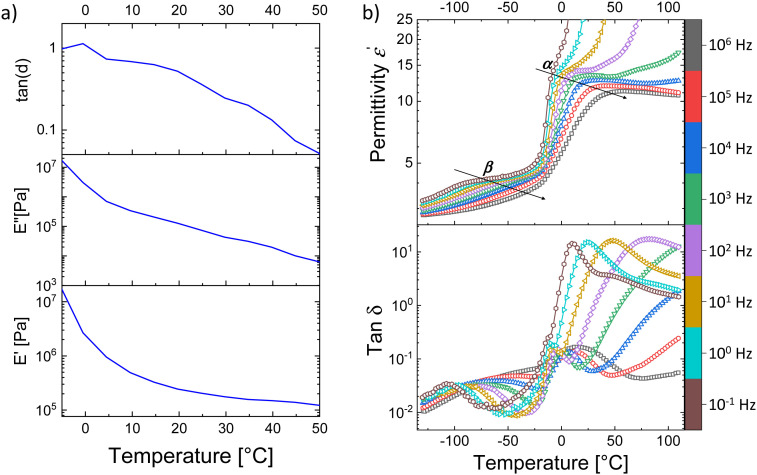
(a) Temperature-dependent DMA measurement of E-CL2-3 at 1 Hz and (b) dielectric permittivity (*ε*′) and dissipation loss factor (tan *δ*) of the E-CL2-3 film as a function of temperature at fixed frequencies.

The real and imaginary parts of elastic modulus and the mechanical loss factor tan *δ* of an E-CL2-3 sample from −5 to 50 °C and −70 to 25 °C at a frequency of 1 Hz are shown in Fig. 2a and Fig. S5 (ESI†), respectively. As expected from the DSC curve (Fig. 1b) the *T*_g_ is observed as a step in the endothermic heat flow curve between −15 and 0 °C. In the case of DMA curves, the glass-transition results in a steep increase in the elastic modulus below 0 °C accompanied by a peak in loss around −8 °C and tan *δ* at 0 °C.

The dielectric properties of materials E-CL2-3 and E-CL4-2 were investigated at different frequencies in a wide temperature range from −100 to +100 °C (Fig. S6 (ESI†) and Fig. 1d). Fig. 2b shows the dielectric permittivity *ε*′ and dissipation loss factor tan *δ* of the E-CL2-3 film as a function of temperature at fixed frequencies. Two main relaxation processes, the *α* and *β* processes, are observed. The *α*-process shifts to higher temperature with an increase in frequency (frequency-dependent behavior), which can be assigned to unfreezing the dipoles in the amorphous polymer chains due to the glass transition occurring in this temperature range.^36^ On fitting the *α*-dielectric loss peaks using the Havriliak and Negami (HN) function and the DCALC program developed by Wübbenhorst *et al.*,^37,38^ an Arrhenius plot of the relaxation time *versus* temperature exhibiting Vogel–Fulcher–Tammann (VFT) behavior was obtained confirming the glass-transition process (Fig. S7, ESI†). A *T*_g_ of −14.3 °C was calculated at a relaxation time of 100 s (log *τ* = 2 s),^39^ close to the value obtained by DSC. Due to the high *T*_g_, the permittivity of both materials stayed around 5 at 10 kHz up to −20 °C, because the dipoles are frozen. Above *T*_g_, the mobility of the polymer chains increases significantly, and the dipoles can orient in an electric field and contribute to permittivity. Both samples showed increased permittivity at 20 °C up to 18.4 at 10 kHz (Fig. 1d).

Below the *T*_g_, we observe a *β*-relaxation as a permittivity step with the corresponding loss peaks in the tan *δ* plot exhibiting frequency-dependent behavior. This can be attributed to the localized motions of the side chains containing polar sulphonyl groups. Just above the *α*-relaxation, we observe a steep increase in permittivity, indicating contributions from dc conductivity. Looking into the dissipation factor in the same temperature range above *T*_g_ (Fig. 2b bottom), we observe a relaxation process with high losses (*α*_s_). The high value of permittivity combined with the pronounced increase in conductivity (Fig S6, ESI†) denotes the origin of this relaxation due to real charges inside the material.^40^ However, the conductivity of samples remained relatively low at 5 × 10^−10^ S cm^−1^ at room temperature, which is attractive for applications in DEAs.

**Fig. 3:**
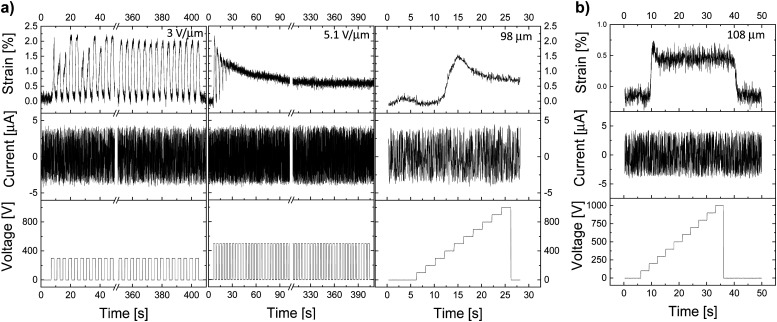
Actuators made of material E-CL2-3 with a thickness of (a) 98 μm and (b) 108 μm. The first actuator (a) was cycled at 300 V and 0.25 Hz for 100 cycles and showed around 2% lateral actuation strain. During the second test at 500 V, the actuation gradually decreased from 2% to 0.75%, and the actuator could not follow the applied voltage at 0.25 Hz. In the third measurement, the voltage was increased every 2 s in a step of 100 V up to 1000 V. The 108-μm thick actuator (b) showed only 0.5% strain at around 200 V, and then the strain was constant until 1000 V.

Electromechanical tests of materials E-CL2-3 and E-CL4-2 were conducted using both thick and thin dielectric films. The typical behavior of actuators constructed from E-CL2-3 thick films can be observed in Fig. 3. They show a small actuation at a low electric field. An increase in the electric field applied to the sample did not increase the actuation but made it irreversible. Additionally, no spike in the leakage current was observed, which suggests that the actuator did not suffer a dielectric breakdown. For instance, a 98-μm thick actuator made of E-CL2-3 showed 2% reversible lateral actuation over 100 cycles at 0.25 Hz and 300 V (3 V μm^−1^) (Fig. 3a). When the voltage was increased to 500 V (5.1 V μm^−1^) at 0.25 Hz, the actuation strain was about 2%, but it diminished within the first few cycles to about 1%. For the next cycles, the actuation was constant at 1% and the actuator did not relax back to the initial shape. The same actuator was subjected to a 100 V step increase every 2 s up to 1000 V (10.2 V μm^−1^). After a small increase in actuation to 1.5% with the applied voltage of 500 V (5.1 V μm^−1^), the actuation slightly decayed and stayed constant at about 1% when further increasing the voltage to 1000 V. No change in the leakage current with increasing voltage was observed. When a 108-μm thick actuator was subjected to the same voltage step increase, again, a small increase in actuation, accompanied by a small decay to a lower but constant actuation, was observed (Fig. 3b). After switching off the voltage, the actuators needed a few seconds to recover the initial shape.

The actuation response of a 101-μm thick actuator made of E-CL2-3 can be found in the ESI† (Fig. S8). Also, this actuator did not show any spike in the leakage current and thus no dielectric breakdown occurred.

**Fig. 4:**
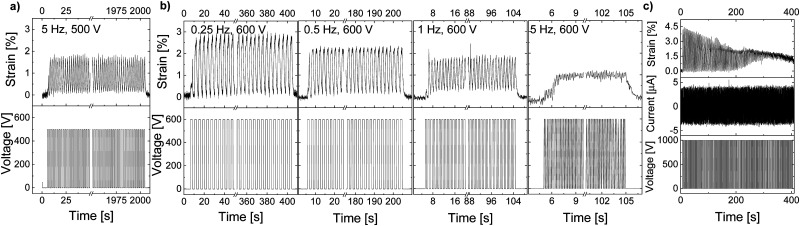
Actuation of a 100-μm thick actuator made of material E-CL4-2: (a) 1000 cycles at 0.5 Hz and 500 V; (b) 100 cycles at 600 V and different frequencies; (c) degradation of the actuation strain at 1000 V and 0.25 Hz within 100 cycles.

Thick actuators made of E-CL4-2 showed a slightly better performance than E-CL2-3 despite the material's higher elastic modulus, which suggested that the dielectric permittivity has a stronger impact on actuation at low electric fields than the mechanical properties. A 100-μm thick actuator displayed almost 2% actuation strain at 500 V (5 V μm^−1^) and 0.5 Hz (Fig. 4a). It also showed a stable actuation over 1000 cycles at 500 V and 0.5 Hz. When the voltage was increased to 600 V, the actuation strain increased to almost 3% at 0.25 Hz but decreased again to 2.3% when increasing the frequency to 0.5 Hz (Fig. 4b). At 1 Hz, the actuation strain showed an even smaller actuation up to around 1.8%, while at 5 Hz, only 1% strain was detected as the actuator apparently did not have sufficient time to relax back to its initial state. The variation in the actuation with the frequency is likely due to the rather strong increase in the mechanical losses with the frequency, which reached 0.57 at 5 Hz. Similar behavior was observed before for the well-explored VHB film.^41,42^ When the voltage was increased to 1000 V (10 V μm^−1^), an actuation of 4% was detected at the beginning. However, within 100 cycles, a significant decrease in actuation strain and a baseline drift were observed (Fig. 4c). This behavior may be due to the ions in the material, which have a higher activation energy than the dipoles and thus need a higher voltage to be polarized. With each applied voltage step, the ions move more toward the electrodes, where probably some irreversible damage of the dielectric or electrode occurs. Similar behavior was observed for another two actuators tested, with a dielectric thickness of 99 μm and 101 μm, respectively (Fig. S9 and S10, ESI†). The higher electric field needed for the irreversible process to set in for E-CL4-2 than for thick E-CL2-3 can be explained by the latter's lower elastic modulus and higher ion conductivity, which will allow ion mobility at lower electric fields.

**Fig. 5:**
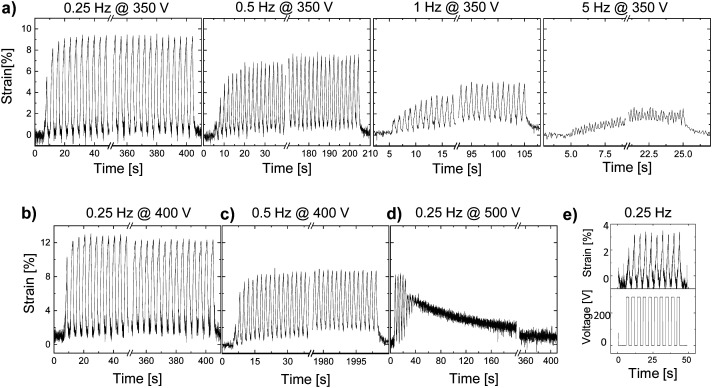
Actuation of a 35-μm thick actuator made of material E-CL2-3: (a) cyclic actuation test at 350 V and different frequencies: 0.25 Hz, 0.5 Hz, 1 Hz, and 5 Hz; (b) 12% actuation strain during 100 cycles at 400 V and 0.25 Hz; (c) 9% actuation strain within 1000 cycles at 400 V and 0.5 Hz; (d) deterioration of actuation after increasing the voltage to 500 V and 0.25 Hz; (e) actuation at 300 V showed that the actuator did not recover its initial performance.

Unlike thick films, thin-film actuators exhibited much better performance with a larger actuation strain. An actuator made of material E-CL2-3 with a thickness of 35 μm showed a 9.5% stable actuation strain over 100 cycles at 350 V (10 V μm^−1^) and 0.25 Hz but exhibited a strongly frequency-dependent response (Fig. 5a). The actuation at 350 V decreased from 9.5% to 7.5%, 4.5%, and 2% when the frequency increased from 0.25 Hz to 0.5 Hz, 1 Hz, and 5 Hz, respectively. The decay in actuation with increasing frequency is explained by the large mechanical losses observed with increasing frequency (Fig. 1c). The largest actuation strain of 12% at 11.4 V μm^−1^ was detected at a frequency of 0.25 Hz and 400 V (Fig. 5b). At 0.5 Hz and 400 V, the actuator showed a stable actuation of 9% over 1000 cycles (Fig. 5c). However, when the voltage was increased to 500 V (14.3 V μm^−1^), the actuation deteriorated after a few cycles (Fig. 5d). This behavior was also observed in thick films, but at lower electric fields. The differences observed in the electric field required for this process to set in for films with different thicknesses may be explained by the change in the mechanical properties in the actuators when biaxially prestrained. Irrespective of film's thickness, the actuators were prestrained by 5%. Therefore, the impact of the prestrain will be different for thin and thick films. Stiffer films of the same material have a lower ionic conductivity. After this test, the actuator was subjected to 10 cycles at 0.25 Hz and 300 V, but only a small actuation of 3% was observed (Fig. 5e), which suggests that the actuators suffered some irreversible degradation, although the electric field at which this degradation occurred was significantly below the dielectric breakdown strength of 24 V μm^−1^ of the material (Fig. S12, ESI†). An actuator made from a 33-μm thin film of E-CL2-3 showed an even larger actuation strain and withstood higher voltages (Fig. S11, ESI†). However, the overall response was similar. The largest actuation of 14% at 800 V and 0.25 Hz was measured for this actuator, which decreased to 7% when increasing the frequency of operation to 5 Hz. However, not all thin-film actuators made of material E-CL2-3 showed good performance, likely due to defects (not shown). As expected, due to the higher elastic modulus of E-CL4-2, thin films gave a lower actuation than E-CL2-3 (Fig. S13, ESI†).

**Fig. 6:**
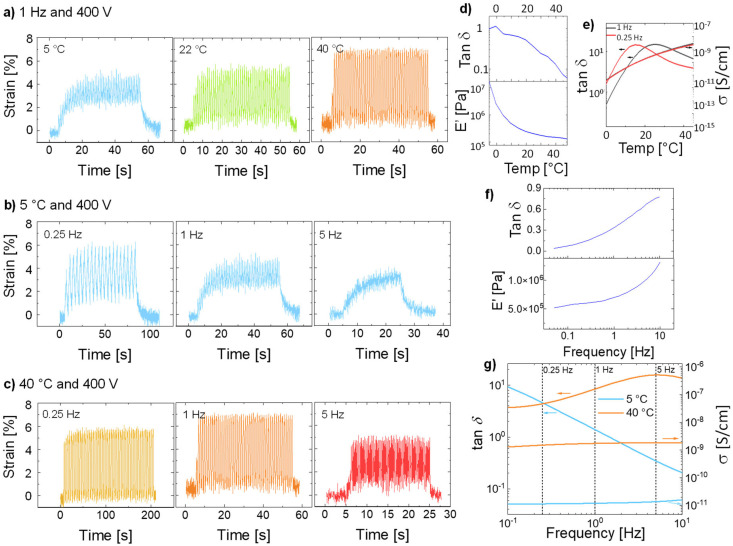
A 36-μm thick actuator made of E-CL2-3 tested at 400 V and (a) at 1 Hz at 5 °C for 50 cycles, at RT for 50 cycles, and 40 °C for 50 cycles; (b) at 5 °C and 0.25 Hz for 20 cycles, 1 Hz for 50 cycles, at 5 Hz for 100 cycles; (c) at 40 °C at 0.25 Hz for 50 cycles, at 1 Hz for 50 cycles, at 5 Hz for 100 cycles. (d) Temperature-dependent DMA measurement of E-CL2-3 at 1 Hz. (e) Temperature-dependent impedance spectroscopy at 0.25 and 1 Hz of E-CL2-3. (f) Frequency-dependent DMA at room temperature of E-CL2-3. (g) tan *δ* and conductivity of E-CL2-3 at frequencies from 0.1 Hz to 1 Hz at 5 and 40 °C.

The reason behind the frequency-dependent actuation response can be seen in the *T*_g_ of the materials and the large mechanical losses at increasing frequencies (Fig. 1c), which are higher than typical materials reported previously by our group, but in the same range as the well-known VHB foil.^27,29,31,42,43^ The actuators were tested only 30 °C above the *T*_g_. This temperature was too close to the *T*_g_, which impaired chains’ mobility. Therefore, we decided to investigate how the temperature influenced the actuation. Fig. 6 shows the behavior of E-CL2-3 (36 μm thick) tested at temperatures from 5 to 40 °C and 400 V and at different frequencies. When the actuator was actuated at 400 V and 1 Hz, but at different temperatures of 5, 22, and 40 °C, a clear increase in the actuation strain with the temperature can be seen (Fig. 6a). This improvement can be explained by the huge change in the storage modulus and the strong decrease in the mechanical losses with increasing temperature (Fig. 6d). The mechanical losses are highest at *T*_g_, which was observed at 0 °C in DMA measurement (Fig. S5, ESI†). The ion conductivity increases with the temperature; thus, if the conductivity negatively affects actuation, the actuation at 40 °C should be affected. However, the actuator shows large and reversible deformation. The mechanical losses increase with increasing frequency and the material was stiffer (Fig. 6f). This change strongly impacts the actuation at high frequency and 5 °C, which is close to the *T*_g_ = 0 °C from the DMA measurement (Fig. 6b). The dielectric losses at 5 °C decrease with the frequency, while the conductivity does not change much (Fig. 6g). Thus, it can be concluded that the actuation response at low temperature and different frequencies is dominated by the mechanical properties of the material. When the temperature was increased to 40 °C, the material reached the rubbery plateau. At this temperature and low frequency, the mechanical losses are low and the material is soft. Therefore, an increase in the actuation strain is observed (Fig. 6c). Again, the conductivity of this material at 40 °C and from 0.1 to 10 Hz does not change much. The decay in actuation at 40 °C with increasing frequency is due to increased mechanical losses and stiffness (Fig. 6f).

**Fig. 7:**
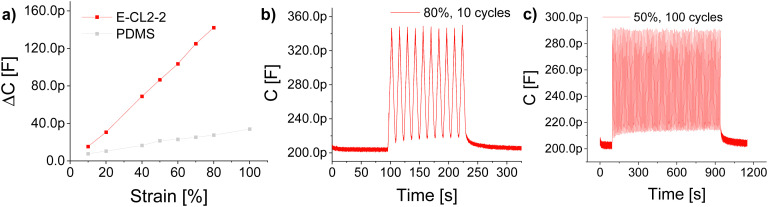
(a) Change in capacitance with strain for E-CL2-2 and PDMS. (b) The change in capacitance for E-CL2-2 at 80% strain over 10 cycles. (c) Stability test for the E-CL2-2 sensor at 50% strain over 100 cycles.

High dielectric permittivity elastomers also allow for increased sensitivity of capacitive sensors.^44–46^ For this application, it is attractive to use elastomers with a higher tensile strength. The mechanical losses are less critical in actuator application as the operation frequency of such sensors is typically 1 Hz, and thus, the materials have sufficient time to relax back to the initial shape. Also the dielectric losses are less critical as the voltage used for operation is 1 V. Therefore, material E-CL2-2 with the highest tensile strength serves the best for such an application. The sensors consisted of a thin layer of E-CL2-2 covered on both sides with cross-linked Elastosil® electrodes. The active sensing area (where the two electrodes overlap) had dimensions of 25 × 10 × 0.2 mm. The VHB™ 4910 film from 3 M was used to insulate the sensor and increase its mechanical stability. For comparison, also a sensor having PDMS as a dielectric was used. Each sensor was cycled 10 times at different strains of 10, 20, 40, 50, 60, 70, and 80%. From each measurement, the average capacitance at a defined strain was taken. The average capacitance in unstrained form was subtracted from the capacitance value of the strained sensor to give Δ*C*. Fig. 7a shows the Δ*C versus* applied strain. The slopes of sensors made of E-CL2-2 and PDMS were 1.83 pF and 0.29 pF, respectively. An enhancement of more than six times in sensitivity is observed for E-CL2-2. As shown in Fig. 7b, the sensor gave a stable and reversible response over 10 cycles at 80% strain. Additionally, the sensor was subjected to 100 cycles at 50% strain (Fig. 7c). It showed a stable change in capacitance within the whole measurement from around 215 pF in the relaxed form to 290 pF in the strained form.

## Conclusions

We have shown that polysiloxane-bearing methyl sulfonyl side groups could be successfully cross-linked into thin-film elastomers using a thiol–ene addition reaction. An elastomer with permittivity as high as 18.4 at 10 kHz and 20 °C was obtained, while the conductivity value was in the range of 5 × 10^−10^ S cm^−1^. The best actuator showed an actuation of 14% at 24.2 V μm^−1^ and 0.25 Hz, while at 18.2 V μm^−1^ and 1 Hz, it had a stable actuation of 8%. Due to the high *T*_g_ = −13.6 °C, the material exhibited a strongly frequency-dependent actuation, giving an almost two times reduction in actuation strain when the frequency was increased from 0.25 Hz to 5 Hz. This reduction can be explained by the strong change in the mechanical properties with the frequency. During the actuation tests at different temperatures, it was found that at a temperature of 40 °C, almost the same actuation strain at 5 Hz was achieved at 5 °C and a lower frequency of 0.25 Hz. Additionally, the material showed promising results as a dielectric in capacitive sensors, with a six times higher sensitivity than conventional silicone rubber and stable capacitance change over 100 cycles at 50% strain. The large change in Δ*ε*′ at near room temperature and the good dielectric and mechanical properties of these materials make them interesting candidates for thermal and mechanical energy harvesting, which should be explored in the future.

## Author contributions

Y. S. performed the synthesis and characterization of all materials. T. R. V. conducted the dielectric breakdown strength, DMA, and impedance spectroscopy measurements. Y. S. and D. M. O. wrote the original draft. D. M. O. initiated the activity, designed the materials, received funding acquisition, and coordinated and supervised this research. All authors contributed to discussions, reviewing, and editing, and have approved the final version of the manuscript.

## Conflicts of interest

There are no conflicts to declare.

## Supplementary Material

TC-011-D3TC00200D-s001
