# Supplementary material for: Synthesis of polysiloxane elastomers modified with sulfonyl side groups and their electromechanical response
Source: J Mater Chem C Mater. 2023 May 18;11(22):7367–76. doi: 10.1039/d3tc00200d (PMC10249063; doi:10.1039/d3tc00200d)
Supplement: TC-011-D3TC00200D-s001 [file TC-011-D3TC00200D-s001.pdf]

Electronic Supplementary Material (ESI) for Journal of Materials Chemistry C.
This journal is © The Royal Society of Chemistry 2023

Supporting Information

Synthesis of polysiloxane elastomers modified with sulfonyl side groups and their electromechanical response

Yauhen Sheima,[a,b] Thulasinath Raman Venkatesan,[a] Holger Frauenrath,[b] and Dorina M. Opris*[a]

[a] *Laboratory for Functional Polymers Swiss Federal Laboratories for Materials Science and Technology Empa Überlandstrasse 129, Dübendorf CH-8600, Switzerland, email: Dorina.opris@empa.ch*

[b] *Institute of Chemical Sciences and Engineering Ecole Polytechnique Federale de Lausanne (EPFL) Station 6, Lausanne CH-1015, Switzerland*

† Electronic Supplementary Information (ESI) available: [details of any supplementary information available should be included here]. See DOI: 10.1039/x0xx00000x

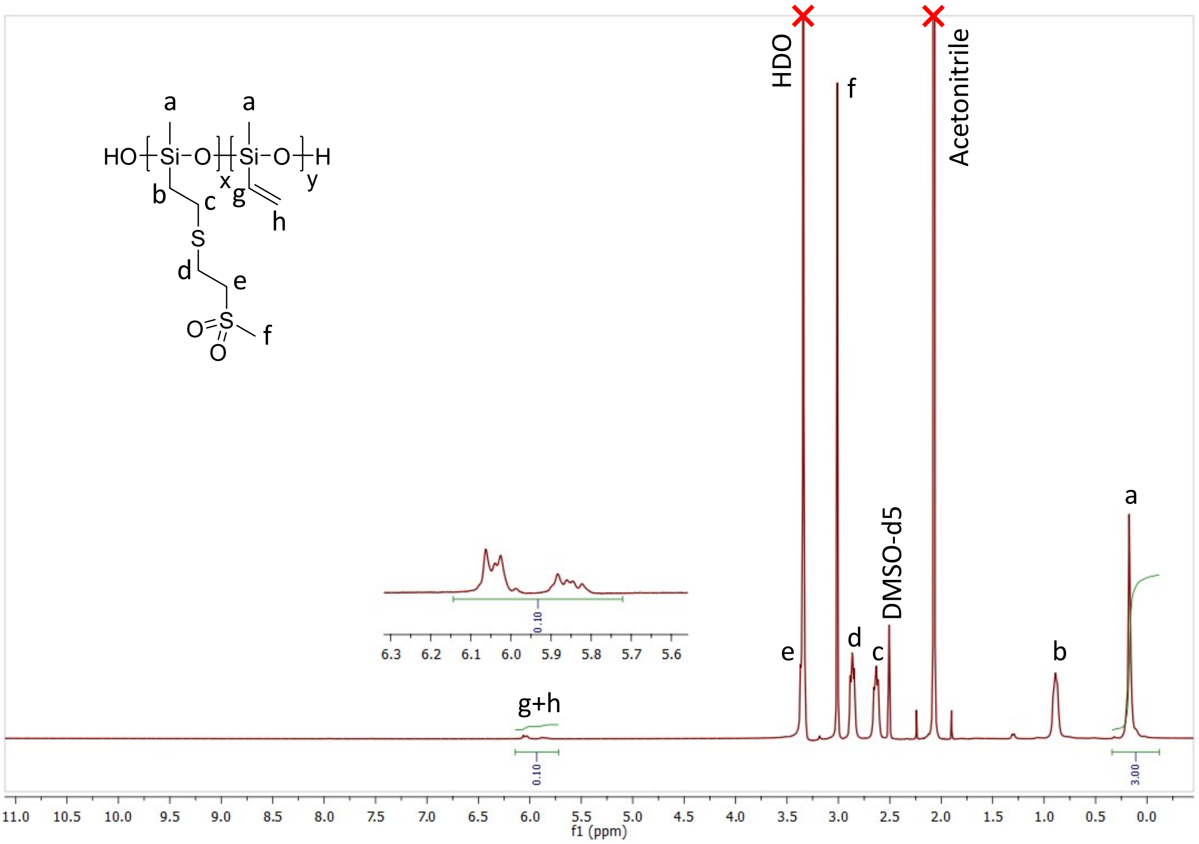

Figure S1. ^{1}H NMR spectrum of polysiloxane functionalized with 2-(methylsulfonyl)-ethanethiol side groups **PSu** (60 wt% in acetonitrile).

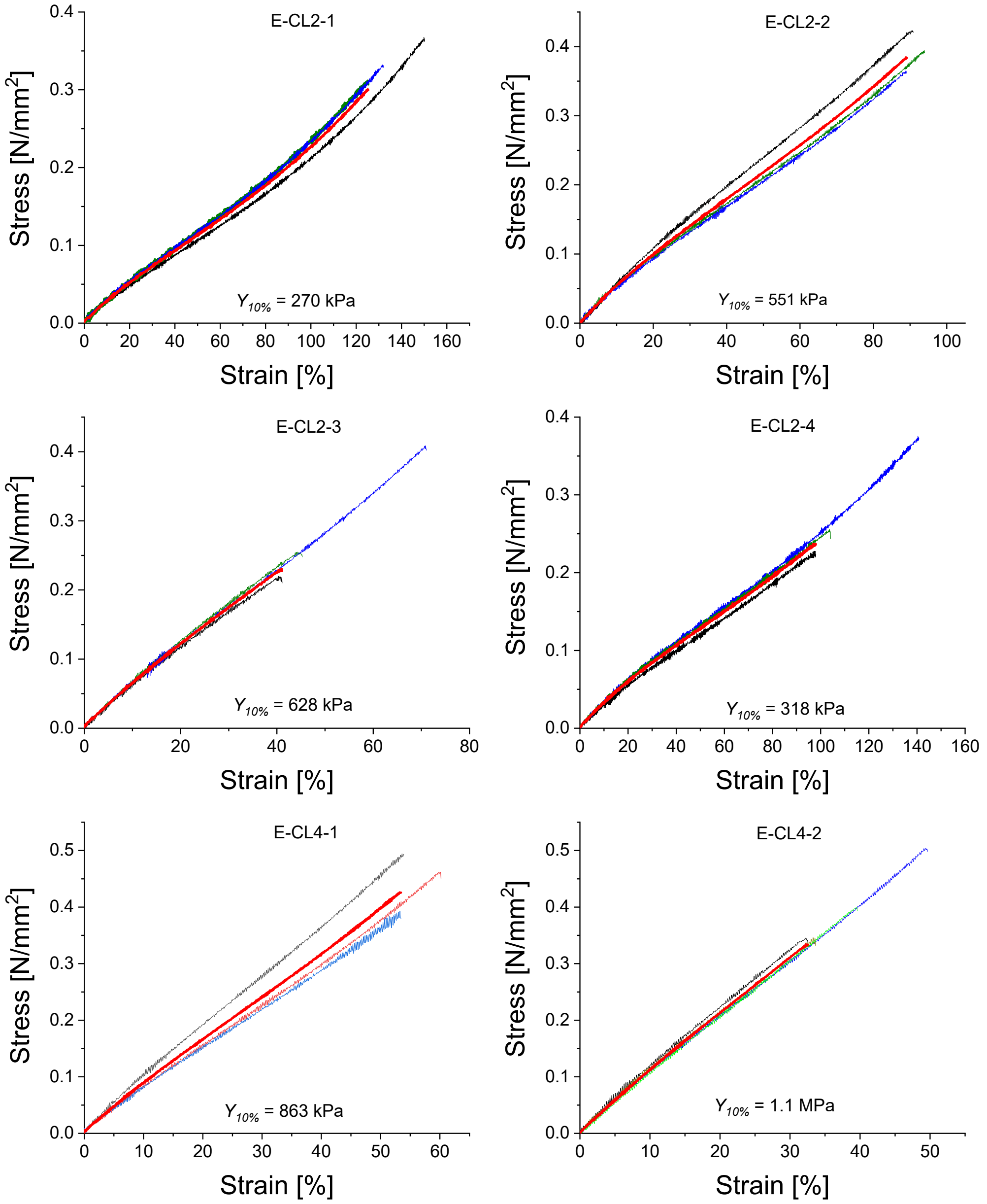

Figure S2. Stress-strain curves of elastomers **E-CLx-Y**. The red curve in each graph is the average of several measurements.

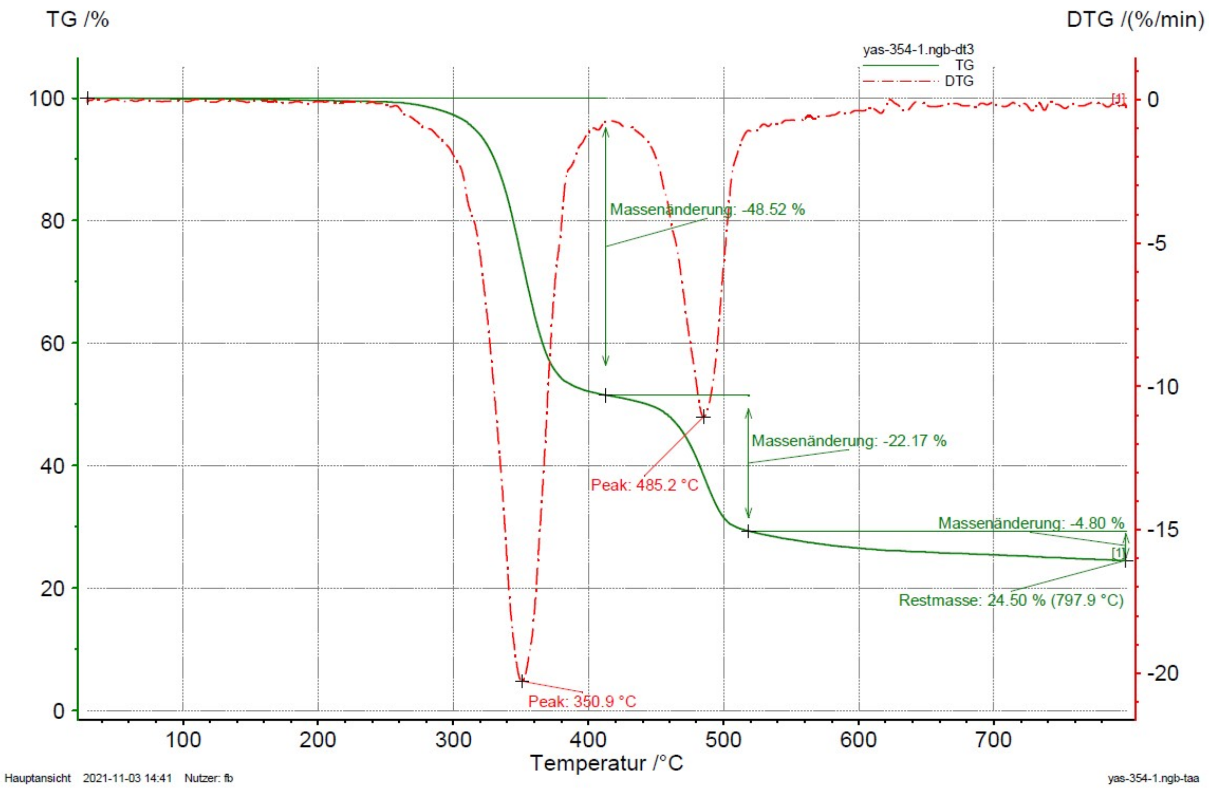

Figure S3. TGA curve of **E-CL2-3**

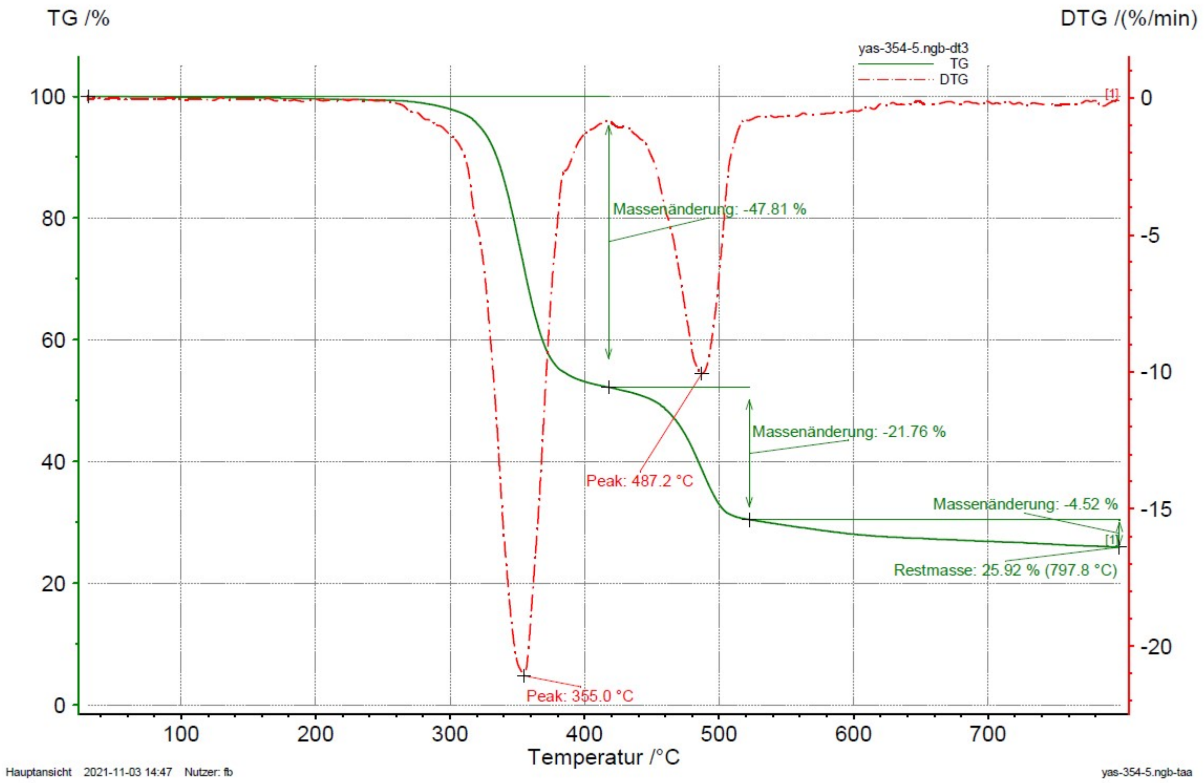

Figure S4. TGA curve of **E-CL4-2**.

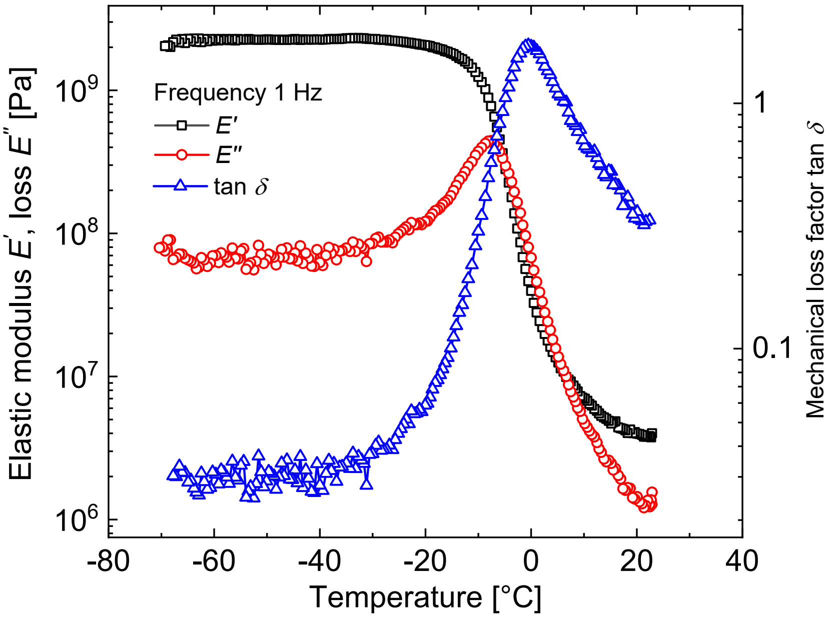

Figure S5. DMA measurement of **E-CL4-2** from -70 to 20 °C at 1 Hz. The peak in tan δ at 0 °C represents the glass transition temperature.

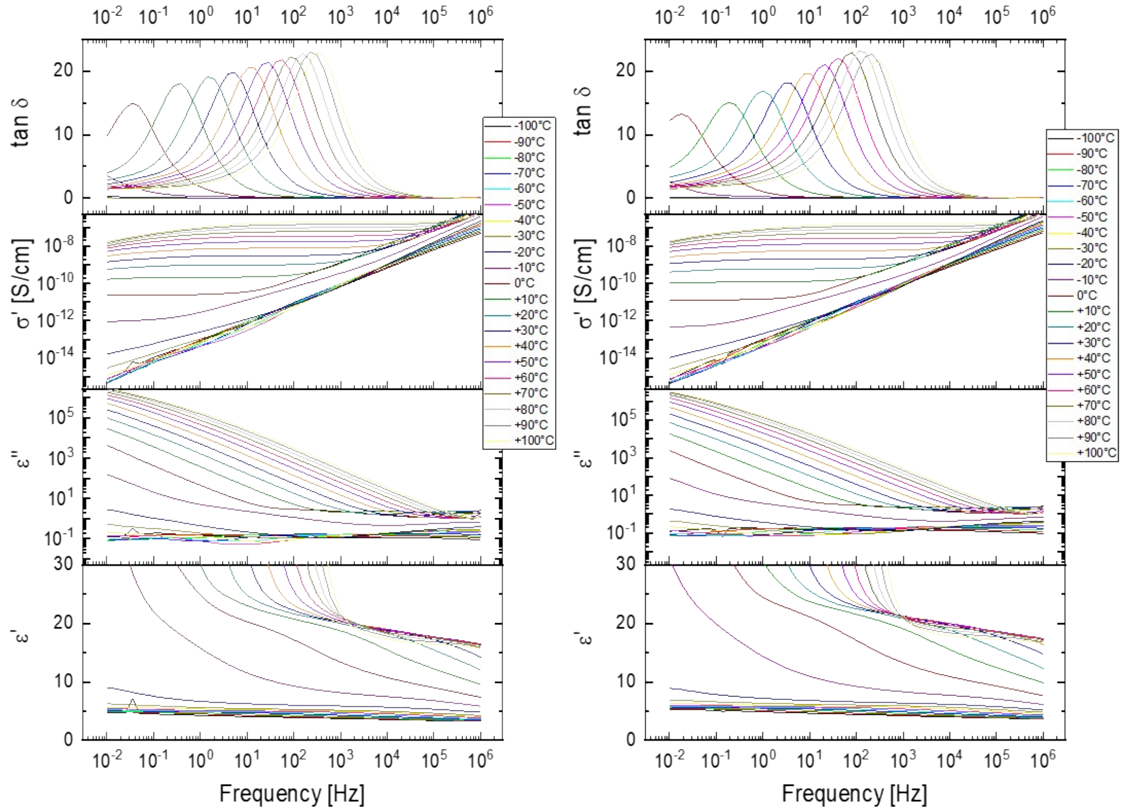

Figure S6. Dielectric permittivity (ε′), dielectric loss (ε″), conductivity (σ'), and tanδ of **E-CL2-3** (left) and **E-CL4-2** (right) at different temperatures and frequencies.

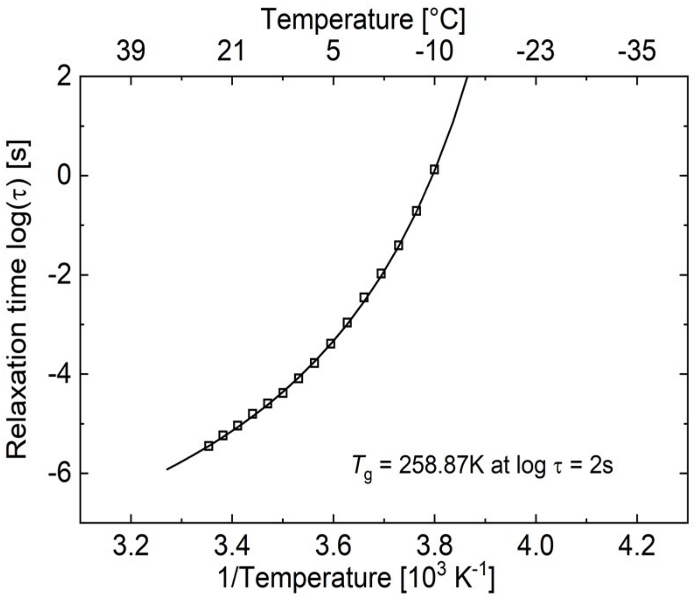

Figure S7. Arrhenius plot of the relaxation time versus temperature exhibiting Vogel-Fulcher-Tamman (VFT) behavior. A T_g of -14.3 °C, close to the DSC value, was calculated at a relaxation time of 100 s (log τ = 2 s).

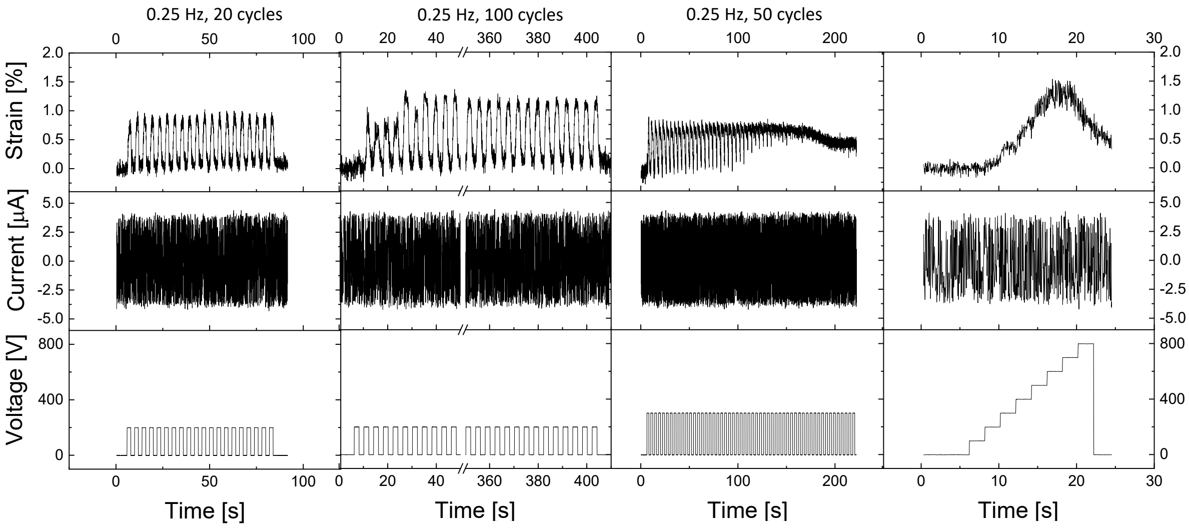

Figure S8. Actuator is made of material **E-CL2-3** with a thickness of 101 μm. Four of more than ten measurements are presented, which describe the typical behavior of this actuator. First, the measurements were done at 200 V. When voltage was increased to 300 V, deterioration of actuation happened. Subsequent application of 800 V didn't result in a higher actuation strain.

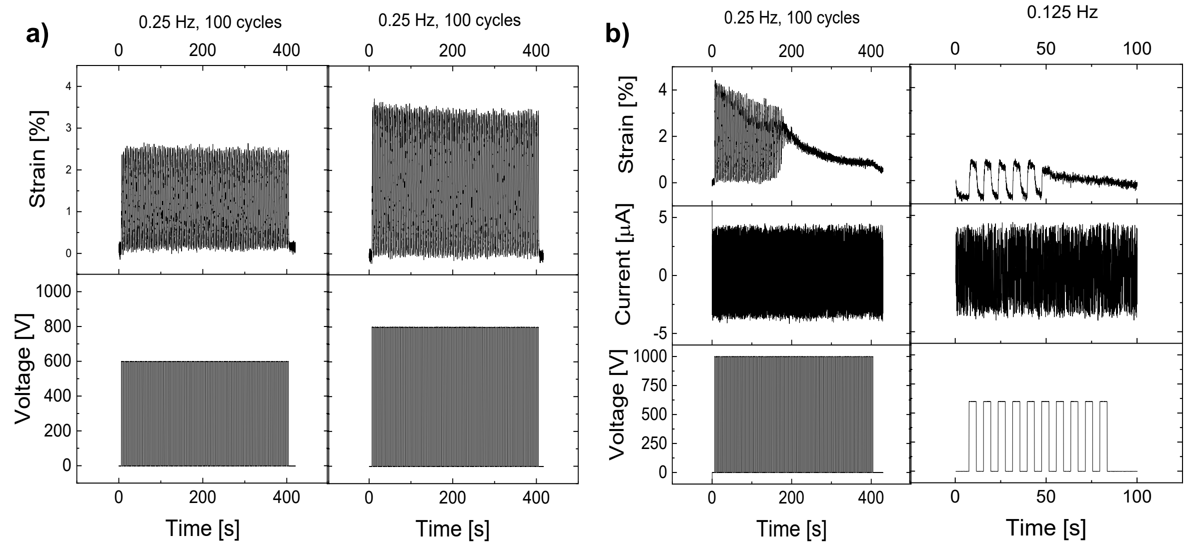

Figure S9. Actuation of a 99 μm thick actuator made of material **E-CL4-2**: a) stable actuation over 100 cycles at 600 V and 800 V and 0.25 Hz; b) degradation of actuation during the 100 cycles test at 1000 V and 0.25 Hz, and subsequent bad actuation at 600 V and 0.125 Hz.

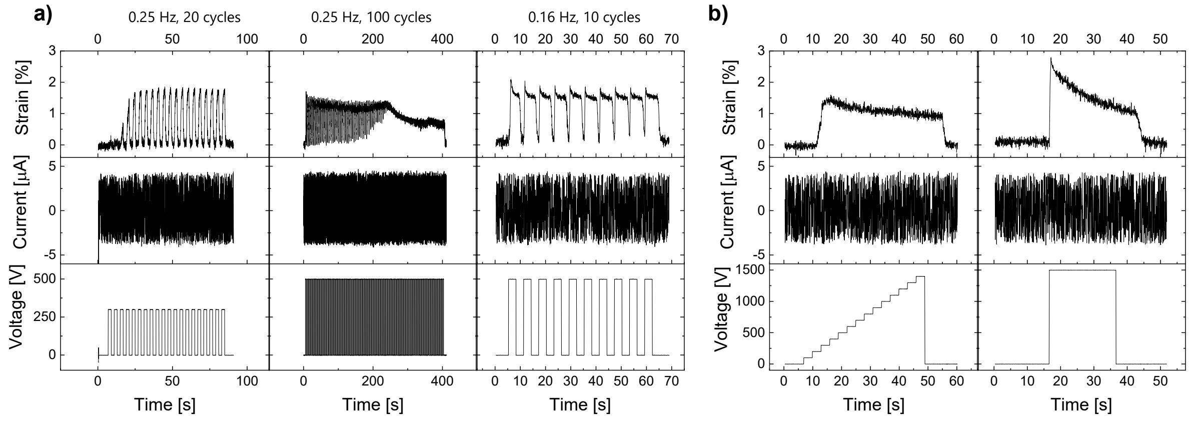

Figure S10. Actuation of a 101 μm thick actuator made of material **E-CL4-2**: a) a 1.8% strain is detected at 300 V and 0.25 Hz. Increasing the voltage to 500 V resulted in a deterioration of actuation. Subsequent application of 500 V at lower frequency (0.16 Hz) resulted in 1.8% actuation, but it can be seen that the actuator doesn't have enough time to relax back to the initial state; b) step voltage increase measurement up to 1500 V and a constant application of 1500 V withing 20 s.

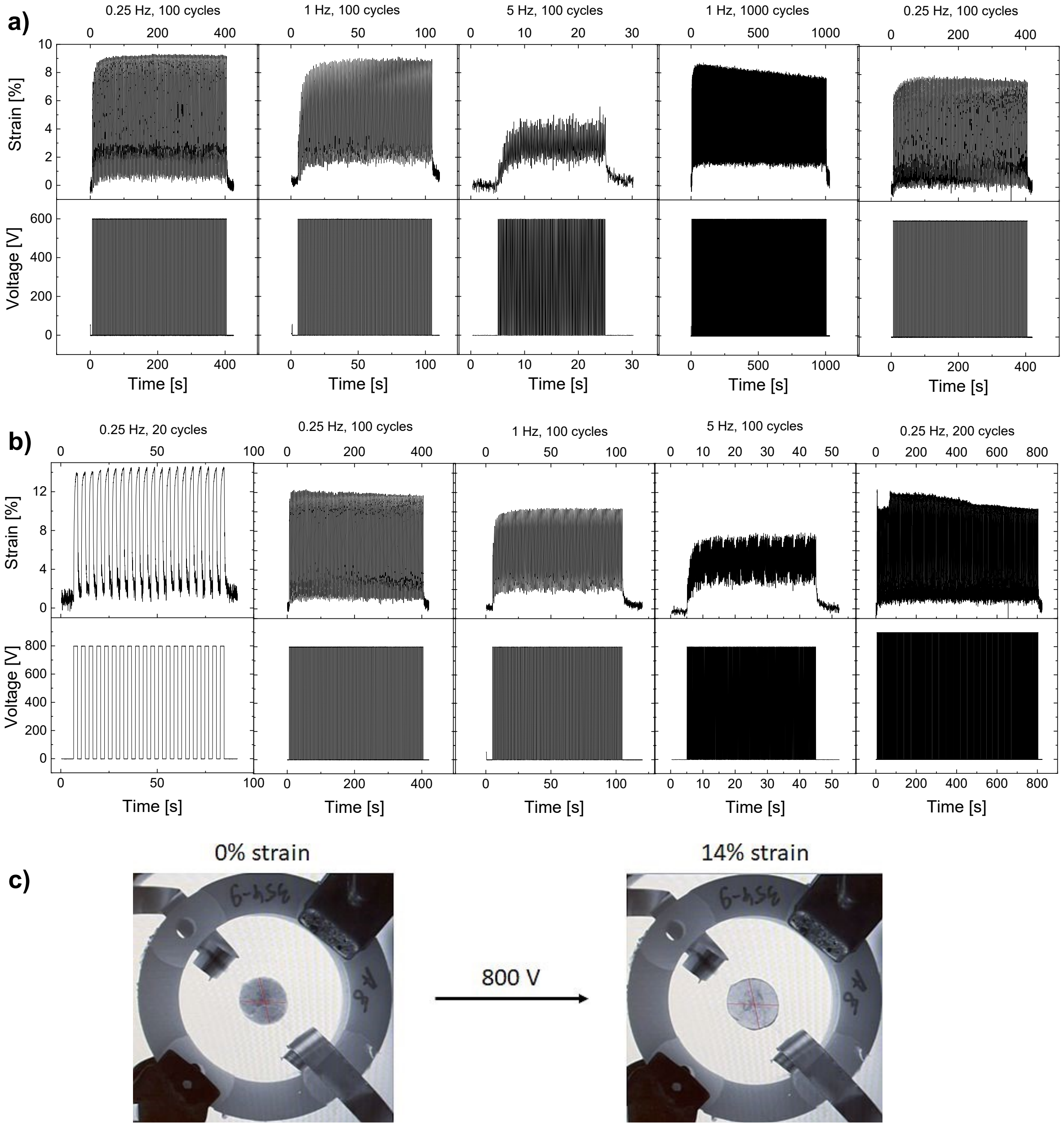

Figure S11. Actuation of a 33 μm thick actuator made of material **E-CL2-3**: a) actuation at 600 V and different frequencies; b) actuation at 800 V and different frequencies; c) photo of the actuator in relaxed and actuated (800 V, 0.25 Hz, 20c) states.

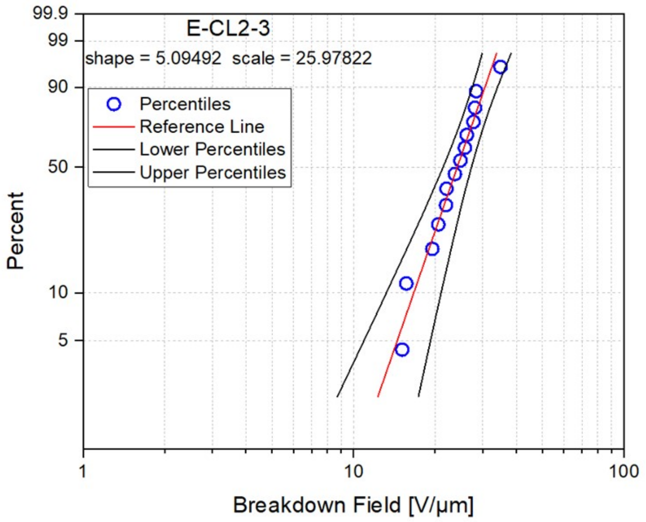

Figure S12. Weibull probability plot of breakdown field for materials **E-CL2-3**. The breakdown strength was tested by placing the material between two metallic electrodes of 1 mm^2 and gradually increasing the voltage until the breakdown was reached. At least ten samples with thicknesses in the range of 100-130 μm were tested. The average breakdown strength was 24 V μm^{-1}.

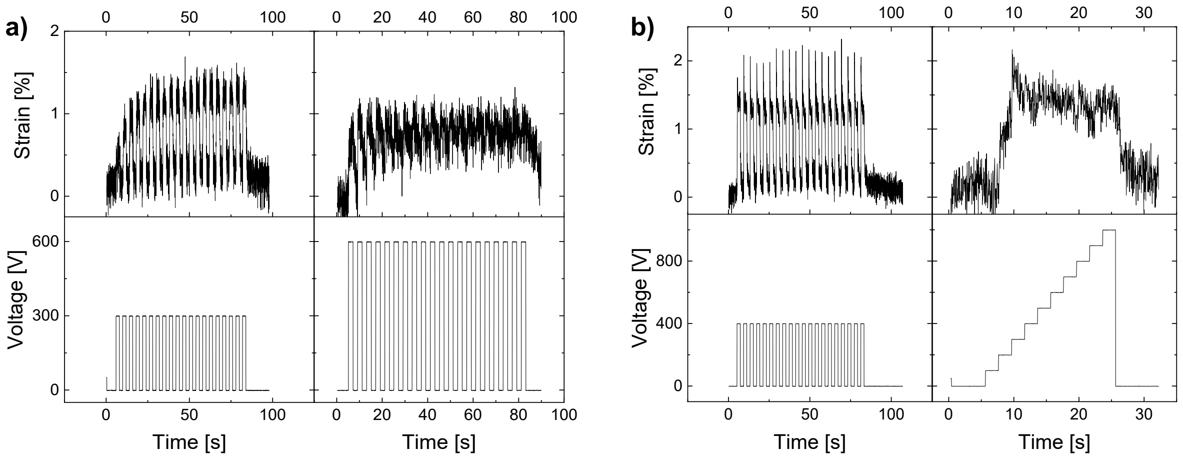

Figure S13. Actuation response of **E-CL4-2**: a) 34 μm film; b) 33 μm film.
